# mGem: Applying microbiome therapeutic learnings to next-generation agricultural bioproducts

**DOI:** 10.1128/mbio.03323-25

**Published:** 2026-02-25

**Authors:** Eric C. Holmes

**Affiliations:** 1 Department of Chemical and Biological Engineering, University of Idaho 5640, Moscow, Idaho, USA; The Ohio State University, Columbus, Ohio, USA

**Keywords:** microbiome therapeutics, plant microbiome, agricultural bioproducts, immune engineering, colonization, biocontrol, cross-kingdom synergy

## Abstract

Biological discoveries in plant and human systems have long advanced our understanding of how signaling, metabolism, and immunity shape cross-kingdom interactions. Building on this rich history of interdisciplinary insight, there is now a tremendous opportunity to strengthen connections between human and agricultural microbiome research. This perspective highlights key biological synergies across these systems that are essential for advancing human, agricultural, and ecosystem health. Focus is given to colonization, immune, and biosafety engineering strategies developed for microbiome therapeutics that can guide the design and development of next-generation agricultural bioproducts. Ultimately, greater knowledge exchange and collaboration across disciplines will be critical to translate microbiome discoveries into bioproducts with positive societal impact.

## PERSPECTIVE

New technologies that leverage the plant microbiome to restore soils, reduce crop loss, and augment fertilizers promise to improve the productivity and efficiency of our food systems (1–3). These living bioproducts, however, face several challenges related to potency, longevity, and regulation that have limited their real-world impacts (2, 4, 5). Many of these hurdles parallel those in microbiome therapeutics, where sustained investment in mechanistic and clinical studies has illuminated strategies for improved microbial function, persistence, and biosafety (6–8). Just as foundational discoveries in innate immunity (9–11), hormone signaling (12), and RNA interference (13, 14) have advanced innovation across plant and human biology, there is now opportunity for reciprocal knowledge exchange between researchers in clinical and agricultural microbiome sciences. Greater integration of approaches for (i) designing enhanced microbiome colonization, (ii) engineering immunomodulatory microbes, and (iii) incorporating biocontrol strategies for health and environmental safety will accelerate progress in both domains, ultimately leading to more effective microbial bioproducts to improve human health and agricultural productivity.

## DESIGNING ENHANCED COLONIZATION

Lessons from clinical microbiome therapeutics provide insight into how to overcome one of the greatest challenges facing both fields: ensuring stable colonization and persistence within complex microbial ecosystems (4, 15). Natural microbial communities are genetically and functionally diverse, and numerous studies have demonstrated their resilience to inoculation with exogenous strains or communities (15–22). This ecological resistance has directly contributed to the failure of promising clinical candidates, motivating the field to investigate the determinants of colonization and to refine both drug and trial designs to maximize engraftment and clinical success (15, 16, 21).

At the core of this challenge lies niche exclusion—the ecological principle that once a well-adapted organism occupies a niche, it is exceedingly difficult to supplant without a significant environmental perturbation (4, 15, 23, 24). A common strategy to overcome this barrier in the clinic is to transiently disrupt the native microbiome using antibiotics and/or bowel preparation, thereby creating open niches that can be occupied by therapeutic strains (15, 25–30). In contrast, broad antibiotic use in agricultural systems is increasingly untenable due to its negative impacts on soil and environmental health (31–33). Consequently, alternative strategies are needed to promote niche generation and sustained colonization in complex plant-associated microbiomes.

**Fig 1:**
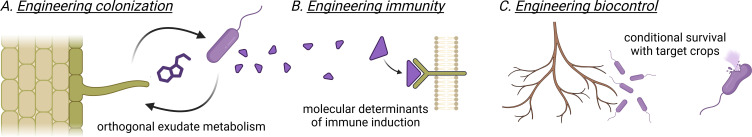
Key opportunities for cross-disciplinary microbiome collaboration include (**A**) engineering colonization using orthogonal carbon substrates, (**B**) engineering immunity using molecular determinants of immune induction, and (**C**) engineering biocontrol with conditional survival circuits.

New engineering approaches for improved colonization should draw inspiration from advances in both microbiome therapeutics and native plant-microbe biology. One promising direction is to exploit the metabolic determinants of colonization which drive community assembly in mammalian and plant systems (Fig. 1A) (34, 35). In the therapeutics space, researchers have enhanced engraftment by engineering the metabolism of porphyrin, an orthogonal dietary molecule that creates a metabolic niche uniquely accessible to a therapeutic strain (36, 37). This proof-of-principle offers a compelling model for agriculture, where analogous orthogonal niches can occur naturally. For example, in the well-characterized *Agrobacterium* pathosystem, pathogen-derived enzymes synthesize unique opines in the plant host, effectively generating an orthogonal nutritional niche to support bacterial growth (38).

As we have learned more about the molecular determinants of plant-microbe symbioses, it has become clear that the ability to metabolize unique plant root exudates provides bacteria with a competitive advantage by enabling niche occupation and colonization (39–46). As we invest efforts into discovering more plant-chemical-microbe interactions, greater emphasis should be placed on leveraging them to design and deploy enhanced colonization strategies (Fig. 1A). Ultimately, advancements in high-throughput screening methods, discovery of new microbial catabolic pathways, and improved synthetic biology tools for non-model organisms will enable approaches to enhance the stability and efficacy of agricultural bioproducts (4, 36, 37).

## ENGINEERING IMMUNOMODULATORY MICROBES

Many microbiome technologies rely upon delicate biochemical and signaling interactions with host immune systems (4, 47). Through these interactions, mammalian microbiomes play a central role in shaping immune development, reducing infection risk, and modulating immune-related diseases (48–52). These host-microbe interactions have provided the conceptual foundation for numerous microbiome therapeutic candidates targeting autoimmune and oncology indications (25, 53–62). Collectively, these efforts demonstrate that microbial biochemistry can be harnessed and engineered to modulate host immunity and address disease.

This principle has important parallels in agriculture. Although plants lack an adaptive immune system, they possess innate immune networks that share striking similarities to those of mammals (9–11), offering a promising framework for synergistic innovation. Proof-of-principle studies in mammalian systems have established that engineering precise molecular mechanisms (e.g., production of unique peptidoglycan fragments in *Enterococcus* [63] or extracellular polysaccharides in *Bacteroides* [64–66]) can shift immune signaling to ameliorate disease. Similar principles should guide efforts to characterize and design disease-suppressive soils and microbes that trigger induced systemic resistance in plants (67, 68). As additional molecular mechanisms that underlie such beneficial phenomena are discovered, evidence-based strategies to enhance these interactions should inform the design of products that improve plant health outcomes (Fig. 1B).

Antigen-specific engineering strategies pioneered in cancer immunotherapy could likewise inspire the next generation of plant-microbe communication tools (57, 61, 69, 70). These foundational studies demonstrate that immune systems can recognize engineered microbial epitopes, which is a broadly applicable principle that can be exploited for agriculture. Researchers are already expanding the sensing capabilities of plant immune receptors through protein engineering, opening the door to orthogonal signaling systems in which plants and beneficial microbes exchange designed molecular cues (71–74). Such systems could coordinate colonization, activate plant defenses, or regulate nutritional exchanges, ultimately expanding the functional scope and efficacy of microbial bioproducts in agriculture.

## INCORPORATING BIOCONTROL STRATEGIES

Designing safe and controllable microbial systems will be critical to realizing the promise of living bioproducts in the clinic and the field. While the testing and approval of genetically modified (GM) microbes are currently governed by separate agencies in the United States, there is growing momentum toward a unified framework and coordinating office (75). Merging these efforts could streamline oversight, strengthen biosafety standards, and help circumvent regulatory discordances akin to those faced by GM crops (75–78). Because human and plant microbiomes are interconnected, shared stewardship and containment approaches will also be vital for ensuring long-term ecosystem health (79, 80).

In clinically tested biotherapeutics, strategies to restrict cell replication, such as attenuation or engineered nutrient auxotrophies, have traditionally dominated (58, 61, 62, 81–85). These approaches are effective in clinical contexts where repeated dosing is feasible, but they are poorly suited to agricultural systems where persistence and colonization are often essential for efficacy and economic viability. Emerging synthetic biocontrol systems, however, offer more adaptable alternatives. These include conditional survival circuits and environment-specific dependencies that allow microbial growth only under defined host or environmental conditions (Fig. 1C) (36, 37, 86–88). Translating these strategies to agriculture would enable designs where engineered plants provide essential metabolites or molecular cues that permit colonization exclusively within the rhizosphere of targeted crops. Such context-dependent control systems would help balance safety with the stable colonization that is necessary for translational success.

Minimizing the chance of horizontal gene transfer is another critical aspect of biocontrol. Both clinical and field studies have demonstrated that heterologous genetic payloads can be transferred to other species once released into natural environments (37, 89, 90). To mitigate this risk, engineered bioproducts should be designed such that transferred genetic material cannot persist or cause ecological harm. Strategies to do so include integrating genetic payloads chromosomally to reduce mobilization (75, 91), recoding genes to prevent expression outside of the intended host (92–94), and coupling functional genes with biocontrol systems that preclude survival in non-target organisms (95, 96). Ultimately, stacking multiple safeguards may be necessary to prevent genetic escape and ensure the evolutionary stability of engineered bioproducts. As clinical and agricultural products progress, a shared regulatory framework, alongside these advanced microbial and genetic strategies for biocontrol, will be essential for developing effective and safe microbial technologies.

## FUTURE SYNERGISTIC OPPORTUNITIES

Bridging design principles from clinic to field offers an opportunity to develop microbial bioproducts that persist, communicate, and function predictably in coordination with their hosts (Fig. 1). Across both domains, success hinges on overcoming a shared set of challenges: promoting colonization within competitive microbial ecosystems, exploiting host immune responses, and implementing reliable biocontrol strategies to ensure safety and stability. These challenges are increasingly convergent, and leveraging discoveries from each field will accelerate innovation in the other.

As agricultural bioproducts continue to advance, new opportunities for field-to-clinic translational learning will emerge. Distinct features of plant-microbe systems, such as the ability to engineer synergistic host-microbe partnerships (73) and the use of controlled-environment facilities, enable in-depth interrogation of host-microbe signaling and long-term evaluation of biocontrol strategies (97, 98). Insights from these systems can, in turn, guide the development of safer, more effective microbiome therapeutics for human health.

Taken together, these reciprocal advances motivate a unified microbiome engineering framework that integrates mechanistic biology, ecological design, and biocontrol principles across domains. Merging approaches from clinical microbiome therapeutics and agricultural biotechnology will empower the design of microbial systems that function predictably within their ecological and host contexts. Through this shared framework, translational microbiome science can move beyond disciplinary boundaries and toward living bioproducts that promote improved human health and agricultural productivity.

## References

[1] Compant S, Cassan F, Kostić T, Johnson L, Brader G, Trognitz F, Sessitsch A. 2025. Harnessing the plant microbiome for sustainable crop production. Nat Rev Microbiol 23:9–23. doi:10.1038/s41579-024-01079-1 39147829

[2] Qiu Z, Egidi E, Liu H, Kaur S, Singh BK. 2019. New frontiers in agriculture productivity: optimised microbial inoculants and in situ microbiome engineering. Biotechnol Adv 37:107371. doi:10.1016/j.biotechadv.2019.03.010 30890361

[3] Trivedi P, Leach JE, Tringe SG, Sa T, Singh BK. 2020. Plant-microbiome interactions: from community assembly to plant health. Nat Rev Microbiol 18:607–621. doi:10.1038/s41579-020-0412-1 32788714

[4] Russ D, Fitzpatrick CR, Teixeira PJPL, Dangl JL. 2023. Deep discovery informs difficult deployment in plant microbiome science. Cell 186:4496–4513. doi:10.1016/j.cell.2023.08.035 37832524

[5] Sergaki C, Lagunas B, Lidbury I, Gifford ML, Schäfer P. 2018. Challenges and approaches in microbiome research: from fundamental to applied. Front Plant Sci 9:1205. doi:10.3389/fpls.2018.01205 30174681 PMC6107787

[6] Charbonneau MR, Isabella VM, Li N, Kurtz CB. 2020. Developing a new class of engineered live bacterial therapeutics to treat human diseases. Nat Commun 11:1738. doi:10.1038/s41467-020-15508-1 32269218 PMC7142098

[7] Bai X, Huang Z, Duraj-Thatte AM, Ebert MP, Zhang F, Burgermeister E, Liu X, Scott BM, Li G, Zuo T. 2023. Engineering the gut microbiome. Nat Rev Bioeng 1:665–679. doi:10.1038/s44222-023-00072-2

[8] Sorbara MT, Pamer EG. 2022. Microbiome-based therapeutics. Nat Rev Microbiol 20:365–380. doi:10.1038/s41579-021-00667-9 34992261

[9] Dardick C, Ronald P. 2006. Plant and animal pathogen recognition receptors signal through Non-RD kinases. PLoS Pathog 2:e2. doi:10.1371/journal.ppat.0020002 16424920 PMC1331981

[10] Nürnberger T, Brunner F, Kemmerling B, Piater L. 2004. Innate immunity in plants and animals: striking similarities and obvious differences. Immunol Rev 198:249–266. doi:10.1111/j.0105-2896.2004.0119.x 15199967

[11] Ausubel FM. 2005. Are innate immune signaling pathways in plants and animals conserved? Nat Immunol 6:973–979. doi:10.1038/ni1253 16177805

[12] Weyers JDB, Paterson NW. 2001. Plant hormones and the control of physiological processes. New Phytol 152:375–407. doi:10.1046/j.0028-646X.2001.00281.x 33862994

[13] Zamore PD. 2006. RNA interference: big applause for silencing in Stockholm. Cell 127:1083–1086. doi:10.1016/j.cell.2006.12.001 17174883

[14] Napoli C, Lemieux C, Jorgensen R. 1990. Introduction of a chimeric chalcone synthase gene into petunia results in reversible co-suppression of homologous genes in trans. Plant Cell 2:279–289. doi:10.1105/tpc.2.4.279 12354959 PMC159885

[15] Porcari S, Benech N, Valles-Colomer M, Segata N, Gasbarrini A, Cammarota G, Sokol H, Ianiro G. 2023. Key determinants of success in fecal microbiota transplantation: from microbiome to clinic. Cell Host Microbe 31:712–733. doi:10.1016/j.chom.2023.03.020 37167953

[16] Landy J, Walker AW, Li JV, Al-Hassi HO, Ronde E, English NR, Mann ER, Bernardo D, McLaughlin SD, Parkhill J, Ciclitira PJ, Clark SK, Knight SC, Hart AL. 2015. Variable alterations of the microbiota, without metabolic or immunological change, following faecal microbiota transplantation in patients with chronic pouchitis. Sci Rep 5:12955. doi:10.1038/srep12955 26264409 PMC4532993

[17] Ericsson AC, Personett AR, Turner G, Dorfmeyer RA, Franklin CL. 2017. Variable colonization after reciprocal fecal microbiota transfer between mice with low and high richness microbiota. Front Microbiol 8. doi:10.3389/fmicb.2017.00196 PMC5322181 28280484

[18] Lozupone CA, Stombaugh JI, Gordon JI, Jansson JK, Knight R. 2012. Diversity, stability and resilience of the human gut microbiota. Nature 489:220–230. doi:10.1038/nature11550 22972295 PMC3577372

[19] McNulty NP, Yatsunenko T, Hsiao A, Faith JJ, Muegge BD, Goodman AL, Henrissat B, Oozeer R, Cools-Portier S, Gobert G, Chervaux C, Knights D, Lozupone CA, Knight R, Duncan AE, Bain JR, Muehlbauer MJ, Newgard CB, Heath AC, Gordon JI. 2011. The impact of a consortium of fermented milk strains on the gut microbiome of gnotobiotic mice and monozygotic twins. Sci Transl Med 3. doi:10.1126/scitranslmed.3002701 PMC3303609 22030749

[20] Cheng AG, Ho P-Y, Aranda-Díaz A, Jain S, Yu FB, Meng X, Wang M, Iakiviak M, Nagashima K, Zhao A, Murugkar P, Patil A, et al.. 2022. Design, construction, and in vivo augmentation of a complex gut microbiome. Cell 185:3617–3636. doi:10.1016/j.cell.2022.08.003 36070752 PMC9691261

[21] Zmora N, Zilberman-Schapira G, Suez J, Mor U, Dori-Bachash M, Bashiardes S, Kotler E, Zur M, Regev-Lehavi D, Brik RB-Z, et al.. 2018. Personalized gut mucosal colonization resistance to empiric probiotics is associated with unique host and microbiome features. Cell 174:1388–1405. doi:10.1016/j.cell.2018.08.041 30193112

[22] Maldonado-Gómez MX, Martínez I, Bottacini F, O’Callaghan A, Ventura M, van Sinderen D, Hillmann B, Vangay P, Knights D, Hutkins RW, Walter J. 2016. Stable engraftment of Bifidobacterium longum AH1206 in the human gut depends on individualized features of the resident microbiome. Cell Host Microbe 20:515–526. doi:10.1016/j.chom.2016.09.001 27693307

[23] Litvak Y, Bäumler AJ. 2019. The founder hypothesis: a basis for microbiota resistance, diversity in taxa carriage, and colonization resistance against pathogens. PLoS Pathog 15:e1007563. doi:10.1371/journal.ppat.1007563 30789972 PMC6383860

[24] Lee SM, Donaldson GP, Mikulski Z, Boyajian S, Ley K, Mazmanian SK. 2013. Bacterial colonization factors control specificity and stability of the gut microbiota. Nature 501:426–429. doi:10.1038/nature12447 23955152 PMC3893107

[25] Baruch EN, Youngster I, Ben-Betzalel G, Ortenberg R, Lahat A, Katz L, Adler K, Dick-Necula D, Raskin S, Bloch N, et al.. 2021. Fecal microbiota transplant promotes response in immunotherapy-refractory melanoma patients. Science 371:602–609. doi:10.1126/science.abb5920 33303685

[26] LaPlante K, Stevens R, Gonzales-Luna AJ. 2024. Systematic review of the orally administered microbiome therapeutic, fecal microbiota spores, live-brpk, to prevent recurrence of Clostridioides difficile infection in adults. SAGE Open Med 12:20503121241274192. doi:10.1177/20503121241274192 39386261 PMC11462573

[27] Louie T, Golan Y, Khanna S, Bobilev D, Erpelding N, Fratazzi C, Carini M, Menon R, Ruisi M, Norman JM, Faith JJ, Olle B, Li M, Silber JL, Pardi DS. 2023. VE303, a defined bacterial consortium, for prevention of recurrent Clostridioides difficile infection: a randomized clinical trial. JAMA 329:1356–1366. doi:10.1001/jama.2023.4314 37060545 PMC10105904

[28] Ianiro G, Punčochář M, Karcher N, Porcari S, Armanini F, Asnicar F, Beghini F, Blanco-Míguez A, Cumbo F, Manghi P, Pinto F, Masucci L, Quaranta G, De Giorgi S, Sciumè GD, Bibbò S, Del Chierico F, Putignani L, Sanguinetti M, Gasbarrini A, Valles-Colomer M, Cammarota G, Segata N. 2022. Variability of strain engraftment and predictability of microbiome composition after fecal microbiota transplantation across different diseases. Nat Med 28:1913–1923. doi:10.1038/s41591-022-01964-3 36109637 PMC9499858

[29] Podlesny D, Durdevic M, Paramsothy S, Kaakoush NO, Högenauer C, Gorkiewicz G, Walter J, Fricke WF. 2022. Identification of clinical and ecological determinants of strain engraftment after fecal microbiota transplantation using metagenomics. Cell Rep Med 3:100711. doi:10.1016/j.xcrm.2022.100711 35931074 PMC9418803

[30] Li M, Xu R, Li Y. 2020. Sequential laxative-probiotic usage for treatment of irritable bowel syndrome: a novel method inspired by mathematical modelling of the microbiome. Sci Rep 10:19291. doi:10.1038/s41598-020-75225-z 33168839 PMC7652883

[31] Batuman O, Britt-Ugartemendia K, Kunwar S, Yilmaz S, Fessler L, Redondo A, Chumachenko K, Chakravarty S, Wade T. 2024. The use and impact of antibiotics in plant agriculture: a review. Phytopathology 114:885–909. doi:10.1094/PHYTO-10-23-0357-IA 38478738

[32] Du L, Liu W. 2012. Occurrence, fate, and ecotoxicity of antibiotics in agro-ecosystems. A review. Agron Sustain Dev 32:309–327. doi:10.1007/s13593-011-0062-9

[33] McManus PS, Stockwell VO, Sundin GW, Jones AL. 2002. Antibiotic use in plant agriculture. Annu Rev Phytopathol 40:443–465. doi:10.1146/annurev.phyto.40.120301.093927 12147767

[34] Ho P-Y, Nguyen TH, Sanchez JM, DeFelice BC, Huang KC. 2024. Resource competition predicts assembly of gut bacterial communities in vitro. Nat Microbiol 9:1036–1048. doi:10.1038/s41564-024-01625-w 38486074

[35] Schäfer M, Pacheco AR, Künzler R, Bortfeld-Miller M, Field CM, Vayena E, Hatzimanikatis V, Vorholt JA. 2023. Metabolic interaction models recapitulate leaf microbiota ecology. Science 381:eadf5121. doi:10.1126/science.adf5121 37410834

[36] Shepherd ES, DeLoache WC, Pruss KM, Whitaker WR, Sonnenburg JL. 2018. An exclusive metabolic niche enables strain engraftment in the gut microbiota. Nature 557:434–438. doi:10.1038/s41586-018-0092-4 29743671 PMC6126907

[37] Whitaker WR, Russ ZN, Stanley Shepherd E, Popov LM, Louie A, Lam K, Zong DM, Gill CCC, Gehrig JL, Rishi HS, Tan JA, Buness A, Godoy J, Banta D, Jaidka S, Wilson K, Flood J, Bukshpun P, Yocum R, Cook DN, Warsi T, McLean L, Sonnenburg JL, DeLoache WC. 2025. Controlled colonization of the human gut with a genetically engineered microbial therapeutic. Science 389:303–308. doi:10.1126/science.adu8000 40674481 PMC13308544

[38] González-Mula A, Lang J, Grandclément C, Naquin D, Ahmar M, Soulère L, Queneau Y, Dessaux Y, Faure D. 2018. Lifestyle of the biotroph Agrobacterium tumefaciens in the ecological niche constructed on its host plant. New Phytol 219:350–362. doi:10.1111/nph.15164 29701262

[39] Finkel OM, Salas-González I, Castrillo G, Conway JM, Law TF, Teixeira P, Wilson ED, Fitzpatrick CR, Jones CD, Dangl JL. 2020. A single bacterial genus maintains root growth in a complex microbiome. Nature 587:103–108. doi:10.1038/s41586-020-2778-7 32999461 PMC10329457

[40] Okutani F, Hamamoto S, Aoki Y, Nakayasu M, Nihei N, Nishimura T, Yazaki K, Sugiyama A. 2020. Rhizosphere modelling reveals spatiotemporal distribution of daidzein shaping soybean rhizosphere bacterial community. Plant Cell Environ 43:1036–1046. doi:10.1111/pce.13708 31875335

[41] Shimasaki T, Masuda S, Garrido-Oter R, Kawasaki T, Aoki Y, Shibata A, Suda W, Shirasu K, Yazaki K, Nakano RT, Sugiyama A. 2021. Tobacco root endophytic Arthrobacter harbors genomic features enabling the catabolism of host-specific plant specialized metabolites. mBio 12:e00846-21. doi:10.1128/mBio.00846-21 34044592 PMC8262997

[42] Hemmerle L, Maier BA, Bortfeld-Miller M, Ryback B, Gäbelein CG, Ackermann M, Vorholt JA. 2022. Dynamic character displacement among a pair of bacterial phyllosphere commensals in situ. Nat Commun 13:2836. doi:10.1038/s41467-022-30469-3 35595740 PMC9123166

[43] Huang AC, Jiang T, Liu Y-X, Bai Y-C, Reed J, Qu B, Goossens A, Nützmann H-W, Bai Y, Osbourn A. 2019. A specialized metabolic network selectively modulates Arabidopsis root microbiota. Science 364:eaau6389. doi:10.1126/science.aau6389 31073042

[44] Nakayasu M, Takamatsu K, Kanai K, Masuda S, Yamazaki S, Aoki Y, Shibata A, Suda W, Shirasu K, Yazaki K, Sugiyama A. 2023. Tomato root-associated Sphingobium harbors genes for catabolizing toxic steroidal glycoalkaloids. mBio 14:e00599-23. doi:10.1128/mbio.00599-23 37772873 PMC10653915

[45] Thoenen L, Kreuzer M, Pestalozzi C, Florean M, Mateo P, Züst T, Wei A, Giroud C, Rouyer L, Gfeller V, Notter MD, Knoch E, Hapfelmeier S, Becker C, Schandry N, Robert CAM, Köllner TG, Bruggmann R, Erb M, Schlaeppi K. 2024. The lactonase BxdA mediates metabolic specialisation of maize root bacteria to benzoxazinoids. Nat Commun 15:6535. doi:10.1038/s41467-024-49643-w 39095376 PMC11297187

[46] Savka MA, Farrand SK. 1997. Modification of rhizobacterial populations by engineering bacterium utilization of a novel plant-produced resource. Nat Biotechnol 15:363–368. doi:10.1038/nbt0497-363 9094139

[47] Shi N, Li N, Duan X, Niu H. 2017. Interaction between the gut microbiome and mucosal immune system. Military Med Res 4:14. doi:10.1186/s40779-017-0122-9 PMC5408367 28465831

[48] Iliev ID, Blander JM, Collins N, Guo C-J, Longman RS, Sonnenberg GF, Zeng MY, Artis D. 2025. Microbiota-mediated mechanisms of mucosal immunity across the lifespan. Nat Immunol 26:1645–1659. doi:10.1038/s41590-025-02281-w 40957908 PMC12700289

[49] Zheng D, Liwinski T, Elinav E. 2020. Interaction between microbiota and immunity in health and disease. Cell Res 30:492–506. doi:10.1038/s41422-020-0332-7 32433595 PMC7264227

[50] Lynch JB, Hsiao EY. 2019. Microbiomes as sources of emergent host phenotypes. Science 365:1405–1409. doi:10.1126/science.aay0240 31604267

[51] Graham DB, Xavier RJ. 2023. Conditioning of the immune system by the microbiome. Trends Immunol 44:499–511. doi:10.1016/j.it.2023.05.002 37236891

[52] Hou K, Wu Z-X, Chen X-Y, Wang J-Q, Zhang D, Xiao C, Zhu D, Koya JB, Wei L, Li J, Chen Z-S. 2022. Microbiota in health and diseases. Sig Transduct Target Ther 7:135. doi:10.1038/s41392-022-00974-4 PMC9034083 35461318

[53] Paramsothy S, Kamm MA, Kaakoush NO, Walsh AJ, van den Bogaerde J, Samuel D, Leong RWL, Connor S, Ng W, Paramsothy R, Xuan W, Lin E, Mitchell HM, Borody TJ. 2017. Multidonor intensive faecal microbiota transplantation for active ulcerative colitis: a randomised placebo-controlled trial. Lancet 389:1218–1228. doi:10.1016/S0140-6736(17)30182-4 28214091

[54] Moayyedi P, Surette MG, Kim PT, Libertucci J, Wolfe M, Onischi C, Armstrong D, Marshall JK, Kassam Z, Reinisch W, Lee CH. 2015. Fecal microbiota transplantation induces remission in patients with active ulcerative colitis in a randomized controlled trial. Gastroenterology 149:102–109. doi:10.1053/j.gastro.2015.04.001 25857665

[55] Silber J, Norman J, Kanno T, Crossette E, Szabady R, Menon R, Marko M, Olle B, Lamousé-Smith E. 2022. Randomized, double-blind, placebo (PBO)-controlled, single- and multiple-dose phase 1 study of VE202, a defined bacterial consortium for treatment of IBD: safety and colonization dynamics of a novel live biotherapeutic product (LBP) in healthy adults. Inflamm Bowel Dis 28:S65–S66. doi:10.1093/ibd/izac015.106 41342324

[56] Kim Y, Kim G, Kim S, Cho B, Kim S-Y, Do E-J, Bae D-J, Kim S, Kweon M-N, Song JS, Park SH, Hwang SW, Kim M-N, Kim Y, Min K, Kim S-H, Adams MD, Lee C, Park H, Park SR. 2024. Fecal microbiota transplantation improves anti-PD-1 inhibitor efficacy in unresectable or metastatic solid cancers refractory to anti-PD-1 inhibitor. Cell Host Microbe 32:1380–1393. doi:10.1016/j.chom.2024.06.010 39059396

[57] Cory L, Chu C. 2014. ADXS-HPV: a therapeutic Listeria vaccination targeting cervical cancers expressing the HPV E7 antigen. Hum Vaccin Immunother 10:3190–3195. doi:10.4161/hv.34378 25483687 PMC4514130

[58] Luke JJ, Piha-Paul SA, Medina T, Verschraegen CF, Varterasian M, Brennan AM, Riese RJ, Sokolovska A, Strauss J, Hava DL, Janku F. 2023. Phase I study of SYNB1891, an engineered E. coli nissle strain expressing STING agonist, with and without atezolizumab in advanced malignancies. Clin Cancer Res 29:2435–2444. doi:10.1158/1078-0432.CCR-23-0118 37227176 PMC11225568

[59] Davar D, Dzutsev AK, McCulloch JA, Rodrigues RR, Chauvin J-M, Morrison RM, Deblasio RN, Menna C, Ding Q, Pagliano O, et al.. 2021. Fecal microbiota transplant overcomes resistance to anti-PD-1 therapy in melanoma patients. Science 371:595–602. doi:10.1126/science.abf3363 33542131 PMC8097968

[60] Routy B, Le Chatelier E, Derosa L, Duong CPM, Alou MT, Daillère R, Fluckiger A, Messaoudene M, Rauber C, Roberti MP, et al.. 2018. Gut microbiome influences efficacy of PD-1–based immunotherapy against epithelial tumors. Science 359:91–97. doi:10.1126/science.aan3706 29097494

[61] Toso JF, Gill VJ, Hwu P, Marincola FM, Restifo NP, Schwartzentruber DJ, Sherry RM, Topalian SL, Yang JC, Stock F, Freezer LJ, Morton KE, Seipp C, Haworth L, Mavroukakis S, White D, MacDonald S, Mao J, Sznol M, Rosenberg SA. 2002. Phase I study of the intravenous administration of attenuated Salmonella typhimurium to patients with metastatic melanoma. J Clin Oncol 20:142–152. doi:10.1200/JCO.2002.20.1.142 11773163 PMC2064865

[62] Hassan R, Alley E, Kindler H, Antonia S, Jahan T, Honarmand S, Nair N, Whiting CC, Enstrom A, Lemmens E, Tsujikawa T, Kumar S, Choe G, Thomas A, McDougall K, Murphy AL, Jaffee E, Coussens LM, Brockstedt DG. 2019. Clinical response of live-attenuated, Listeria monocytogenes expressing mesothelin (CRS-207) with chemotherapy in patients with malignant pleural mesothelioma. Clin Cancer Res 25:5787–5798. doi:10.1158/1078-0432.CCR-19-0070 31263030 PMC8132300

[63] Griffin ME, Espinosa J, Becker JL, Luo J-D, Carroll TS, Jha JK, Fanger GR, Hang HC. 2021. Enterococcus peptidoglycan remodeling promotes checkpoint inhibitor cancer immunotherapy. Science 373:1040–1046. doi:10.1126/science.abc9113 34446607 PMC9503018

[64] Ramakrishna C, Kujawski M, Chu H, Li L, Mazmanian SK, Cantin EM. 2019. Bacteroides fragilis polysaccharide A induces IL-10 secreting B and T cells that prevent viral encephalitis. Nat Commun 10:2153. doi:10.1038/s41467-019-09884-6 31089128 PMC6517419

[65] Nam J, Kim A, Kim K, Moon JH, Baig J, Phoo M, Moon JJ, Son S. 2024. Engineered polysaccharides for controlling innate and adaptive immune responses. Nat Rev Bioeng 2:733–751. doi:10.1038/s44222-024-00193-2 41059472 PMC12499632

[66] Mazmanian SK, Liu CH, Tzianabos AO, Kasper DL. 2005. An immunomodulatory molecule of symbiotic bacteria directs maturation of the host immune system. Cell 122:107–118. doi:10.1016/j.cell.2005.05.007 16009137

[67] Pieterse CMJ, Zamioudis C, Berendsen RL, Weller DM, Van Wees SCM, Bakker PAHM. 2014. Induced systemic resistance by beneficial microbes. Annu Rev Phytopathol 52:347–375. doi:10.1146/annurev-phyto-082712-102340 24906124

[68] Schlatter D, Kinkel L, Thomashow L, Weller D, Paulitz T. 2017. Disease suppressive soils: new insights from the soil microbiome. Phytopathology 107:1284–1297. doi:10.1094/PHYTO-03-17-0111-RVW 28650266

[69] Redenti A, Im J, Redenti B, Li F, Rouanne M, Sheng Z, Sun W, Gurbatri CR, Huang S, Komaranchath M, Jang Y, Hahn J, Ballister ER, Vincent RL, Vardoshivilli A, Danino T, Arpaia N. 2024. Probiotic neoantigen delivery vectors for precision cancer immunotherapy. Nature 635:453–461. doi:10.1038/s41586-024-08033-4 39415001 PMC11560847

[70] Chen YE, Bousbaine D, Veinbachs A, Atabakhsh K, Dimas A, Yu VK, Zhao A, Enright NJ, Nagashima K, Belkaid Y, Fischbach MA. 2023. Engineered skin bacteria induce antitumor T cell responses against melanoma. Science 380:203–210. doi:10.1126/science.abp9563 37053311 PMC12356174

[71] Zhang S, Liu S, Lai H-F, Bender KW, Kim G, Caflisch A, Zipfel C. 2025. Reverse engineering of the pattern recognition receptor FLS2 reveals key design principles of broader recognition spectra against evading flg22 epitopes. Nat Plants 11:1642–1657. doi:10.1038/s41477-025-02050-5 40721668 PMC12364711

[72] Li T, Jarquin Bolaños E, Stevens DM, Sha H, Prigozhin DM, Coaker G. 2025. Unlocking expanded flagellin perception through rational receptor engineering. Nat Plants 11:1628–1641. doi:10.1038/s41477-025-02049-y 40721669 PMC12364713

[73] Boo A, Toth T, Yu Q, Pfotenhauer A, Fields BD, Lenaghan SC, Stewart CN, Voigt CA. 2024. Synthetic microbe-to-plant communication channels. Nat Commun 15:1817. doi:10.1038/s41467-024-45897-6 38418817 PMC10901793

[74] Haskett TL, Paramasivan P, Mendes MD, Green P, Geddes BA, Knights HE, Jorrin B, Ryu M-H, Brett P, Voigt CA, Oldroyd GED, Poole PS. 2022. Engineered plant control of associative nitrogen fixation. Proc Natl Acad Sci USA 119. doi:10.1073/pnas.2117465119 PMC9169844 35412890

[75] Chemla Y, Sweeney CJ, Wozniak CA, Voigt CA. 2025. Design and regulation of engineered bacteria for environmental release. Nat Microbiol 10:281–300. doi:10.1038/s41564-024-01918-0 39905169

[76] George DR, Danciu M, Davenport PW, Lakin MR, Chappell J, Frow EK. 2024. A bumpy road ahead for genetic biocontainment. Nat Commun 15:650. doi:10.1038/s41467-023-44531-1 38245521 PMC10799865

[77] Lucht JM. 2015. Public acceptance of plant biotechnology and GM crops. Viruses 7:4254–4281. doi:10.3390/v7082819 26264020 PMC4576180

[78] Turnbull C, Lillemo M, Hvoslef-Eide TAK. 2021. Global regulation of genetically modified crops amid the gene edited crop boom - a review. Front Plant Sci 12:630396. doi:10.3389/fpls.2021.630396 33719302 PMC7943453

[79] Banerjee S, van der Heijden MGA. 2023. Soil microbiomes and one health. Nat Rev Microbiol 21:6–20. doi:10.1038/s41579-022-00779-w 35999468

[80] Ma H, Cornadó D, Raaijmakers JM. 2025. The soil-plant-human gut microbiome axis into perspective. Nat Commun 16:7748. doi:10.1038/s41467-025-62989-z 40835614 PMC12368272

[81] Brockstedt DG, Giedlin MA, Leong ML, Bahjat KS, Gao Y, Luckett W, Liu W, Cook DN, Portnoy DA, Dubensky TW. 2004. Listeria-based cancer vaccines that segregate immunogenicity from toxicity. Proc Natl Acad Sci USA 101:13832–13837. doi:10.1073/pnas.0406035101 15365184 PMC518841

[82] Kurtz CB, Millet YA, Puurunen MK, Perreault M, Charbonneau MR, Isabella VM, Kotula JW, Antipov E, Dagon Y, Denney WS, Wagner DA, West KA, Degar AJ, Brennan AM, Miller PF. 2019. An engineered E. coli nissle improves hyperammonemia and survival in mice and shows dose-dependent exposure in healthy humans. Sci Transl Med 11:eaau7975. doi:10.1126/scitranslmed.aau7975 30651324

[83] Vockley J, Sondheimer N, Puurunen M, Diaz GA, Ginevic I, Grange DK, Harding C, Northrup H, Phillips JA, Searle S, Thomas JA, Zori R, Denney WS, Ernst SL, Humphreys K, McWhorter N, Kurtz C, Brennan AM. 2023. Efficacy and safety of a synthetic biotic for treatment of phenylketonuria: a phase 2 clinical trial. Nat Metab 5:1685–1690. doi:10.1038/s42255-023-00897-6 37770764

[84] Limaye SA, Haddad RI, Cilli F, Sonis ST, Colevas AD, Brennan MT, Hu KS, Murphy BA. 2013. Phase 1b, multicenter, single blinded, placebo-controlled, sequential dose escalation study to assess the safety and tolerability of topically applied AG013 in subjects with locally advanced head and neck cancer receiving induction chemotherapy. Cancer 119:4268–4276. doi:10.1002/cncr.28365 24114811

[85] Cotton MF, Madhi SA, Luabeya AK, Tameris M, Hesseling AC, Shenje J, Schoeman E, Hatherill M, Desai S, Kapse D, et al.. 2022. Safety and immunogenicity of VPM1002 versus BCG in South African newborn babies: a randomised, phase 2 non-inferiority double-blind controlled trial. Lancet Infect Dis 22:1472–1483. doi:10.1016/S1473-3099(22)00222-5 35772447

[86] Gallagher RR, Patel JR, Interiano AL, Rovner AJ, Isaacs FJ. 2015. Multilayered genetic safeguards limit growth of microorganisms to defined environments. Nucleic Acids Res 43:1945–1954. doi:10.1093/nar/gku1378 25567985 PMC4330353

[87] Chan CTY, Lee JW, Cameron DE, Bashor CJ, Collins JJ. 2016. “Deadman” and “passcode” microbial kill switches for bacterial containment. Nat Chem Biol 12:82–86. doi:10.1038/nchembio.1979 26641934 PMC4718764

[88] Stirling F, Bitzan L, O’Keefe S, Redfield E, Oliver JWK, Way J, Silver PA. 2017. Rational design of evolutionarily stable microbial kill switches. Mol Cell 68:686–697. doi:10.1016/j.molcel.2017.10.033 29149596 PMC5812007

[89] Peters M, Heinaru E, Talpsep E, Wand H, Stottmeister U, Heinaru A, Nurk A. 1997. Acquisition of a deliberately introduced phenol degradation operon, pheBA, by different indigenous Pseudomonas species. Appl Environ Microbiol 63:4899–4906. doi:10.1128/aem.63.12.4899-4906.1997 9406411 PMC168818

[90] Elken E, Heinaru E, Jõesaar M, Heinaru A. 2020. Formation of new PHE plasmids in pseudomonads in a phenol-polluted environment. Plasmid 110:102504. doi:10.1016/j.plasmid.2020.102504 32289323

[91] Moura de Sousa J, Lourenço M, Gordo I. 2023. Horizontal gene transfer among host-associated microbes. Cell Host Microbe 31:513–527. doi:10.1016/j.chom.2023.03.017 37054673

[92] Grome MW, Nguyen MTA, Moonan DW, Mohler K, Gurara K, Wang S, Hemez C, Stenton BJ, Cao Y, Radford F, Kornaj M, Patel J, Prome M, Rogulina S, Sozanski D, Tordoff J, Rinehart J, Isaacs FJ. 2025. Engineering a genomically recoded organism with one stop codon. Nature 639:512–521. doi:10.1038/s41586-024-08501-x 39910296 PMC11903333

[93] Zürcher JF, Robertson WE, Kappes T, Petris G, Elliott TS, Salmond GPC, Chin JW. 2022. Refactored genetic codes enable bidirectional genetic isolation. Science 378:516–523. doi:10.1126/science.add8943 36264827 PMC7614150

[94] Nyerges A, Vinke S, Flynn R, Owen SV, Rand EA, Budnik B, Keen E, Narasimhan K, Marchand JA, Baas-Thomas M, Liu M, Chen K, Chiappino-Pepe A, Hu F, Baym M, Church GM. 2023. A swapped genetic code prevents viral infections and gene transfer. Nature 615:720–727. doi:10.1038/s41586-023-05824-z 36922599 PMC10151025

[95] Blazejewski T, Ho H-I, Wang HH. 2019. Synthetic sequence entanglement augments stability and containment of genetic information in cells. Science 365:595–598. doi:10.1126/science.aav5477 31395784

[96] Chlebek JL, Leonard SP, Kang-Yun C, Yung MC, Ricci DP, Jiao Y, Park DM. 2023. Prolonging genetic circuit stability through adaptive evolution of overlapping genes. Nucleic Acids Res 51:7094–7108. doi:10.1093/nar/gkad484 37260076 PMC10359631

[97] French E, Kaplan I, Iyer-Pascuzzi A, Nakatsu CH, Enders L. 2021. Emerging strategies for precision microbiome management in diverse agroecosystems. Nat Plants 7:256–267. doi:10.1038/s41477-020-00830-9 33686226

[98] Roy J, Rineau F, De Boeck HJ, Nijs I, Pütz T, Abiven S, Arnone JA 3rd, Barton CVM, Beenaerts N, Brüggemann N, et al.. 2021. Ecotrons: powerful and versatile ecosystem analysers for ecology, agronomy and environmental science. Glob Chang Biol 27:1387–1407. doi:10.1111/gcb.15471 33274502 PMC7986626

